# Supplementary material for: Implementing active surveillance for TB—The views of managers in a resource limited setting, South Africa
Source: PLoS One. 2020 Oct 2;15(10):e0239430. doi: 10.1371/journal.pone.0239430 (PMC7531829; doi:10.1371/journal.pone.0239430)
Supplement: S1 File — (PDF) [file pone.0239430.s001.pdf]

Interview guide for TB managers. (SUB STUDY 1)

1. What are the current modes of identifying suspected TB cases in your District?

2. What is your opinion on the current modes of identifying TB suspects in the Eastern Cape?

3. What is your understanding of active surveillance for active TB?

4. What are the challenges currently experienced by WBOTs in providing active surveillance for TB as part of the COPC services in the community?

5. What do you consider the most ideal model for active surveillance of active TB in the Eastern Cape context?

6. Can active surveillance be part of an integrated health care service provided in the community that is focused on health promotion and disease prevention i.e. a Community Oriented Primary Care Service (COPC) e.g. Ward Based Outreach Teams (WBOT)

7. What key elements should be considered in implementing active surveillance for TB in communities in the Eastern Cape?

8. What enablers of active surveillance for active TB in high risk communities exist in the current Eastern Cape context?

9. What barriers for active surveillance for active TB in high risk communities exist in the Eastern Cape context?

10. In performing active surveillance for TB in the Eastern Cape context, how do we measure the efficiency of active surveillance for TB?

11. What is required of teams implementing active surveillance for TB in communities?
