# Supplementary material for: Implementing active surveillance for TB—The views of managers in a resource limited setting, South Africa
Source: PLoS One. 2020 Oct 2;15(10):e0239430. doi: 10.1371/journal.pone.0239430 (PMC7531829; doi:10.1371/journal.pone.0239430)
Supplement: Attachment — Submitted filename: Response to Reviewers.pdf [file pone.0239430.s002.pdf]

Response to Reviewers

Thank you for the very encouraging critique of this paper.

Reviewer 1 comments	Author's response
In the introduction section you should mention that TB is THE LEADING cause of mortality in South Africa<br><br>This section, while very interesting is quite long. I recommend that you try to make this more concise. As noted below, some of the information provided in the introduction describing the programme might be better suited in the methods section.	This is now emphasized more than it was.<br><br>Information on COPC has been moved to the methods and the text also edited to be shorter and more concise.
In the section on the setting you might want to consider providing some information on the burden of TB and the coinciding HIV prevalence.<br><br>Additionally, if you have any information on the average income/housing situation there this might help to better contextualize the context.<br><br>In the section on the data collection, you might want to specify what language the interviews were done in and if they required any translation.<br><br>Additionally, it might be useful to provide some information on the questions asked. You could consider including the questionnaire in an annex. Overall, without being able to review the semi-structured questionnaire it is hard to understand the questions asked and what further areas were investigated. I think that sharing this would help future research carry out similar investigations or make the needed adaptations to them.<br><br>Lines 152-160 could be included under a subheading titled programme description. Then I think some of the details	Paragraph added.<br><br>Done.<br><br>All interviews were conducted in English without translation – added.<br><br>An interview guide used for the semi structured interviews has been submitted as requested. Note however that this is a guide to a qualitative interview and not a questionnaire per se.<br><br>Detail on COPC has been added to the setting and edited to be more concise.

regarding the programme provided in the introduction section could be moved here	
Discussion:<br>I recommend that you include the summary of the key findings in the results section rather than the discussion section. Maybe you could do that in the beginning of the results section and then unpack each point further in the subsections as you have already done. This could for example be placed after line 203.<br><br>In the discussion check your sentence on lines 451-52; it does not make sense.<br>On line 463 is there a more recent document you can reference.<br><br>Paragraphs on 433-441 and 469-482 both talk about security risks and challenges as a result. You might want to consider combining these paragraphs to avoid redundancy.<br><br>On lines 492-499 it seems as though you are discussing some of your imitations. However, I recommend that you specifically stipulate that the study is subject to limitations which include XXXXXXX. You have included all the relevant information here, I just think that it needs to be slightly reframed.	The summary of the results section has been moved into the results section as requested. This is placed at the end of the results section, summarizing findings.<br><br>Lines 451-52 has been adjusted for clarity of meaning.<br><br>Done.<br><br>Done.<br><br>Sign posting is now provided in the discussion section to provide clarity, including for the section on limitations.
Reviewer 2 comments	
The introduction should perform a more thorough literature review to identify gaps in our understanding of how to design active surveillance for TB.	Done.
Did the interviews ask about prioritization of the different innovative ideas? The solutions are presented as a long list, which makes it hard for resource-limited programs to	This study was a qualitative study to explore the different issues and approaches from the perspectives of managers. There was no attempt to quantify, prioritize or rank the themes.

identify the highest-impact components to implement. Is there a way to infer some sort of prioritization from the frequency or intensity of themes raised by interviewees?	This qualitative study will inform a second cross sectional survey that is planned across two districts to quantify the issues identified qualitatively.
Table 1 clearly summarizes the results in an accessible way. I suggest using a framework like this for the rest of the paper. In its current form, the results and discussion sections meander a little through the responses to questions rather than concisely laying out the main themes from the interviews and illustrative quotes to accompany them.<br><br>The conclusion that "this study suggests a multi-faceted facility and community-based approach would be most effective for active case finding" is quite general, especially considering it's based on direct interviews with TB program managers. I suggest combing through the results in this paper to identify a more significant contribution to our knowledge that this study brings to light.	Table 1 has been moved to the results section as a summary of the findings. The results section was read and edited where possible to be more focused and concise.<br><br>The conclusion has been revised as advised.
The quotes included in the article do not add an awful lot of color or information to the paper. I'd suggest including a table with several shorter quotes on each topic that focus on key phrases related to the study themes. Perhaps the discussion could weave more detail around the quotes to make it clear what each quote brings to light.	We have provided interpretation of our data with a few supporting quotes to add credibility to the analysis and support key points. In our view the interpretation provided by the researchers to all their data is more important than the illustrative quotations, which cannot do justice to all of the data. We prefer to keep the quotes in the text where they directly support the key points being made rather than separating them in a table. In our view the discussion should deal with the interpretation of the data as a whole and not specific illustrative quotations.
