# Supplementary material for: Implementing active surveillance for TB—The views of managers in a resource limited setting, South Africa
Source: PLoS One. 2020 Oct 2;15(10):e0239430. doi: 10.1371/journal.pone.0239430 (PMC7531829; doi:10.1371/journal.pone.0239430)
Supplement: Attachment — Submitted filename: Response to Reviewers Sept 2020.docx [file pone.0239430.s003.docx]

Dear reviewers

**Response to Reviewers**

Thank you for the very encouraging critique of this paper.

| **Reviewer comments** | **Author’s response** |
| --- | --- |
| While I see that the authors have adequately responded to most of the reviewers' comments and suggestions and have satisfactorily revised their manuscript.  I particularly noticed that they have not addressed the issue of stigmatizing language and terms, such a defaulters.  I also recommend that authors use the COREQ checklist, or other relevant checklists listed by the Equator Network, such as the SRQR, to ensure complete reporting. | The authors have taken care to remove all stigmatizing language and terms as recommended by the reviewers.  A COREQ checklist has been used to check the completeness of the reporting in this paper and is included for the reviewers. |

Thank you again.

Sincerely,

Authors.
